# A comprehensive phytochemical, biological, and toxicological studies of roots and aerial parts of *Crotalaria burhia* Buch.-Ham: An important medicinal plant

**DOI:** 10.3389/fpls.2022.988352

**Published:** 2022-09-16

**Authors:** Sirajudheen Anwar, Muhammad Faisal Nadeem, Irfan Pervaiz, Umair Khurshid, Nimra Akmal, Khurram Aamir, Muhammad Haseeb ur Rehman, Khaled Almansour, Farhan Alshammari, Mohd Farooq Shaikh, Marcello Locatelli, Nafees Ahemad, Hammad Saleem

**Affiliations:** ^1^Department of Pharmacology and Toxicology, College of Pharmacy, University of Hail, Hail, Saudi Arabia; ^2^Institute of Pharmaceutical Sciences (IPS), University of Veterinary and Animal Sciences (UVAS), Lahore, Pakistan; ^3^Department of Pharmacy, The University of Chenab, Gujrat, Pakistan; ^4^Department of Pharmaceutical Chemistry, Faculty of Pharmacy, The Islamia University of Bahawalpur, Bahawalpur, Pakistan; ^5^Akhtar Saeed College of Pharmacy, Canal Campus, Lahore, Pakistan; ^6^Department of Pharmacology, Faculty of Pharmaceutical Sciences, Government College University, Faisalabad, Pakistan; ^7^Department of Pharmaceutics, College of Pharmacy, University of Hail, Hail, Saudi Arabia; ^8^Jeffrey Cheah School of Medicine and Health Sciences, Neuropharmacology Research Laboratory, Monash University Malaysia, Bandar Sunway, Selangor, Malaysia; ^9^Department of Pharmacy, University “G. d’Annunzio” of Chieti-Pescara, Chieti, Italy; ^10^School of Pharmacy, Monash University Malaysia, Bandar Sunway, Selangor, Malaysia

**Keywords:** *Crotalaria burhia*, secondary metabolites, antioxidant, enzyme inhibition, toxicity

## Abstract

This study was designed to seek the phytochemical analysis, antioxidant, enzyme inhibition, and toxicity potentials of methanol and dichloromethane (DCM) extracts of aerial and root parts of *Crotalaria burhia*. Total bioactive content, high-performance liquid chromatography-photodiode array detector (HPLC-PDA) polyphenolic quantification, and ultra-high performance liquid chromatography-mass spectrometry (UHPLC-MS) analysis were utilized to evaluate the phytochemical composition. Antioxidant [including 2,2-diphenyl-1-picryl-hydrazyl-hydrate (DPPH)], 2,2′-azino-bis[3-ethylbenzothiazoline-6-sulfonic acid (ABTS), ferric reducing antioxidant power assay (FRAP), cupric reducing antioxidant capacity CUPRAC, phosphomolybdenum, and metal chelation assays] and enzyme inhibition [against acetylcholinesterase (AChE), butyrylcholinesterase (BChE), α-glucosidase, α-amylase, and tyrosinase] assays were carried out for biological evaluation. The cytotoxicity was tested against MCF-7 and MDA-MB-231 breast cell lines. The root-methanol extract contained the highest levels of phenolics (37.69 mg gallic acid equivalent/g extract) and flavonoids (83.0 mg quercetin equivalent/g extract) contents, and was also the most active for DPPH (50.04 mg Trolox equivalent/g extract) and CUPRAC (139.96 mg Trolox equivalent /g extract) antioxidant assays. Likewise, the aerial-methanol extract exhibited maximum activity for ABTS (94.05 mg Trolox equivalent/g extract) and FRAP (64.23 mg Trolox equivalent/g extract) assays. The aerial-DCM extract was noted to be a convincing cholinesterase (AChE; 4.01 and BChE; 4.28 mg galantamine equivalent/g extract), and α-glucosidase inhibitor (1.92 mmol acarbose equivalent/g extract). All of the extracts exhibited weak to modest toxicity against the tested cell lines. A considerable quantities of gallic acid, catechin, 4-OH benzoic acid, syringic acid, vanillic acid, 3-OH-4-MeO benzaldehyde, epicatechin, *p*-coumaric acid, rutin, naringenin, and carvacrol were quantified *via* HPLC-PDA analysis. UHPLC-MS analysis of methanolic extracts from roots and aerial parts revealed the tentative identification of important phytoconstituents such as polyphenols, saponins, flavonoids, and glycoside derivatives. To conclude, this plant could be considered a promising source of origin for bioactive compounds with several therapeutic uses.

## Introduction

Plants are genetically very diverse and vital to human existence, shelter, food, and medicine. Among plants, the study of medicinal plants has gained worldwide attention in recent years. A substantial amount of research demonstrates the intriguing potential of medicinal plants employed in traditional, complementary, and alternative methods of treating human ailments (Fitzgerald et al., 2020; Erdinc et al., 2021; Tamer et al., 2021). The investigation of medicinal plants as a unique source of enzyme inhibitors, natural antioxidant components, and treatments for a variety of common illnesses has attracted considerable interest (Phumthum et al., 2018). Phytochemicals, also known as secondary metabolites, are bioactive plant molecules and the source of the majority of currently accessible pharmaceuticals. 77% of antibiotics and 547 medicines approved by the FDA by the end of 2013 were derived from natural products, according to a survey (Patridge et al., 2016). Natural products play a major role in medication development; therefore, screening plants for substantial active ingredients can be viewed as a first step toward producing more effective treatments against a broader range of ailments (Bibi Sadeer et al., 2022). Herbal applications are now a rapidly expanding market, with the goal of creating new pharmaceutical and nutraceutical materials with herbal ingredients. Lifestyle diseases such as obesity, cancer, and diabetes mellitus are to blame for the current state of affairs (Ceylan et al., 2016; Yener et al., 2018).

*Crotalaria* belongs to the family Fabaceae. Approximately 700 species are make up this family disseminated throughout the world’s tropical and subtropical regions (Lewis, 2005). In the desert regions of West Pakistan, India, and Afghanistan, *C. burhia*, or Khip, is found as a shrub and fibrous plant. The ancient Indian Ayurvedic system, identified this plant as having great medicinal potential. Anticancer and soothing properties are found in the leaves, roots, and branches of *C. burhia*, while fresh plant juice can be used to treat eczema, gout, hydrophobia, pain, and edema. Roots extract with sugar is used to alleviate chronic kidney pain and to treat typhoid fever. It has a wide range of medical properties (Talaviya et al., 2018), Cooling medication can be made from the plant’s leaves, branches, and roots. Gout, eczema, hydrophobia, pain and swelling, wounds and cuts, infection, renal pain, stomach disorders, rheumatism, and joint pain can all be treated using plant juice in traditional medicine (Katewa and Galav, 2006; Sandeep et al., 2010; Bibi et al., 2015). There are several active compounds in this plant, including triterpenoids, flavonoids, anthraquinones, phenols, polyphenols, steroids, alkaloids, and tannins (Kataria et al., 2011; Kumar et al., 2011; Bibi et al., 2015). Additionally, *C. burhia’s* antibacterial, anti-inflammatory, and antinociceptive properties are supported by its traditional applications (Kataria et al., 2010; Kataria et al., 2012; Soni, 2014; Talaviya et al., 2014; Bibi et al., 2015). *Crotalaria burhia* is a highly important medicinal plant used to treat different ailments. Some researchers also mentioned that the whole plant, as well as its different parts like its branches, roots, leaves, and stem applied for the cure of diseases (Talaviya et al., 2018). Fresh plant juices have magical ethnobotanical values and are reported to treat different disorders. *Crotalaria burhia* is a valuable plant used to treat cancer, infections, pain, swelling, inflammation, hydrophobia, and skin diseases (Kataria et al., 2010). This plant is well known for the useful cure of general contaminations in the Thal Desert of Punjab (Niaz et al., 2013). Previous literature exposed that it is also utilized as a good soil binder, as food for goats, and in the desert to make sheds for animals and ropes (Soni, 2014). Some phytochemical studies reported the isolation of secondary metabolites from *Crotalaria burhia* are identified as toxicarol, elliptone, rotenone, sumatrol, deguelin, and tephrosin (Uddin and Khanna, 1979), crotalarine (Ali and Adil, 1973), crosemperine (Ahmad and Fatima, 1986), quercetin, β-sitosterol (Soni, 2014). However, many species of the *Crotalaria* genus are yet to be explored scientifically.

Polyphenol compounds, which include flavonoids and phenolic acids, are widely distributed throughout the plant kingdom. Over 6,000 different flavonoid species have been discovered so far. In the fight against microbial and insect attacks, they play an important role (Boǧa et al., 2016; Bouhafsoun et al., 2018; Bakir et al., 2020). The biological activities of *C. burhia*, a species of the *Crotalaria* genus, was examined in this study with regard to enzymes targeted for the treatment of diabetes type II, Alzheimer’s disease, and skin hyperpigmentation problems. Methanol and DCM were used to extract the aerial and root sections of *C. burhia*, and ultra-high performance liquid chromatography-mass spectrometry (UHPLC-MS) profiling, HPLC poly-phenolic quantification, and total bioactive contents were used to determine the phytochemical composition of each extract. Several *in vitro* bio-assays were used to measure the antioxidant capacity of each extract, including the phosphomolybdenum assay, DPPH and ABTS assays for radical scavenging, FRAP and CUPRAC for reducing power, and total antioxidant capacity. The inhibition potential of all the extracts was studied against a panoply of clinically important enzymes, including AChE, BChE, glucosidase, amylase, and tyrosinase. Furthermore, statistical correlation of all the activities by principal component analysis (PCA) was also studied.

## Materials and methods

### Plant material and extraction

Dr. H. Waris, Taxonomist of the Cholistan Institute of Desert Studies, The Islamia University of Bahawalpur, recognized *C. burhia* aerial and root parts obtained from Bahawalpur, Pakistan. For future reference, the herbarium of the Department of Pharmacy and Alternative Medicine, also deposited a voucher specimen number. For 15 days, the plant material was kept in the shade to dry. Using a combination of DCM and methanol, the powdered dried plant was extracted over the course of 72 h and further concentrated using rotary evaporator.

### Phytochemical composition

#### Total bioactive contents

Standard Folin-Ciocalteu and aluminum chloride techniques (Slinkard and Singleton, 1977; Zengin et al., 2016) with minor modifications were used to assess the total phenolic (TPC) and flavonoid (TFC) concentrations. Gallic acid equivalents (mg GAE/g extract) and quercetin equivalents (mg QE/g extract) were used to measure phenolic and flavonoid content, respectively.

#### High-performance liquid chromatography-photodiode array detector polyphenolic quantification

High-performance liquid chromatography-photodiode array detector (HPLC-PDA) analysis was used to determine the presence of 22 distinct polyphenolic standards in each sample. Waters liquid chromatograph with a model 600 solvent pump and a 2996 PDA detector was used for the analysis. The data was collected using Empower v.2 Software (Waters Spa, Milford, MA, United States) (Locatelli et al., 2017). The details of HPLC instrumentation are provided in “Supplementary Material” section. The gradient profiles and calibration parameters of the quantified phenolic standards are provided in Supplementary Tables 1, 2, respectively.

#### Ultra-high performance liquid chromatography-mass spectrometry analysis

RP-UHPLC-MS was used to profile secondary metabolites. An Agilent 6,520 was used to perform UHPLC-MS analysis of methanolic extracts of aerial and root portions (negative ionization mode) on the Agilent 1,290 Infinity LC system (Khurshid et al., 2019). In order to make some tentative predictions about the presence of various secondary metabolites in the samples, we turned to the METLIN database. The details of UHPLC-MS instrumentation are provided in “Supplementary Material” section.

### Biological activities

#### Antioxidant assays

According to already adopted methods by Grochowski et al. (2017), DPPH and ABTS radical scavenging, reducing power (FRAP, CUPRAC), total antioxidant capacity (phosphomolybdenum), and metal chelating power of the investigated extracts were evaluated. The antioxidant activity of all assays was measured in terms of Trolox equivalents (mg TE/g extract) while the metal chelating activity was assessed in terms of mg EDTAE/g extract. The details of antioxidant assays are provided in “Supplementary Material” section.

#### Enzyme inhibition assays

The enzyme inhibition potential of plant extracts against cholinesterases (AChE and BChE), tyrosinase, α-amylase, and α-glucosidase was evaluated using previously established *in vitro* standard methods (Grochowski et al., 2017; Mollica et al., 2017). Galantamine equivalents per gram of extract (GALAE/g) were used to measure AChE and BChE inhibitory activities. On the other hand, millimoles of acarbose equivalents (ACAE/g) and milligram of kojic acid equivalents (KAE/g) were used to measure inhibition of α-amylase, α-glucosidase, and tyrosinase, respectively. The details of enzyme inhibition assays are provided in “Supplementary Material” section.

#### Cytotoxicity assay

Using the previously published approach, the cytotoxicity of the tested products was assessed against two breast cancer cell lines, MDA-MB 231 and MCF-7 cells, using the MTT [3-(4,5-dimethylthiazol-2-yl)-2,5-diphenyltetrazolium bromide] assay (Nemudzivhadi and Masoko, 2014). The cell viability percentage (%) was calculated.

### Statistical analysis

Three separate experiments were conducted for each of the assays. Mean standard deviation was used to express results (SD). SPSS v.17.0 was employed for data analysis. ANOVA and Tukey’s test were used to examine the differences between the means. Statistical significance was defined as a *p*-value of 0.05 or less. A link between bioactive content and evaluated biological assays was obtained using PCA and Pearson linear correlation.

## Results and discussion

### Phytochemical profiling

When it comes to plant secondary metabolites, phytochemicals, such as phenols and flavonoids, are regarded to be the most bioactive secondary metabolites (Rahman et al., 2018). Table 1 lists the TPC and TFC values of methanol and DCM extracts of *C. burhia’s* aerial and root portions, respectively. The methanolic root extract had the highest TPC concentration (37.69 mg GAE/g), whilst the DCM aerial extract had the lowest (27.62 mg GAE/g). The flavonoid content determination followed a similar trend to that of the TPC, with TFC values of 83.11 and 12.64 mg QE/g extract for both methanol root and DCM aerial extracts, respectively.

**TABLE 1:** Total bioactive contents and antioxidant properties of *C. burhia* aerial and root extracts.

Extracts	Total bioactive contents		Antioxidant assays					
	Total phenolic content (mg GAE/g)	Total flavonoid content (mg QE/g)	Radical scavenging activity		Reducing power		Total antioxidant capacity (TAC)	Ferrous chelating
			DPPH (mgTE/g extract)	ABTS (mgTE/g extract)	FRAP (mgTE/g extract)	CUPRAC (mgTE/g extract)	Phosphomolybdenum (mgTE/g extract)	Metal chelating (mgEDTAE/g)
CbA-M	28.35 ± 0.56	21.76 ± 0.83	41.25 ± 0.86	94.05 ± 2.94	64.23 ± 1.74	107.62 ± 3.65	8.60 ± 0.21	1.40 ± 0.06
CbA-D	27.62 ± 1.14	12.64 ± 0.16	21.05 ± 0.48	48.22 ± 0.81	48.54 ± 3.03	106.01 ± 2.75	60.46 ± 1.74	2.24 ± 0.11
CbR-M	37.69 ± 1.13	83.11 ± 0.93	50.04 ± 1.85	86.21 ± 0.93	53.87 ± 1.81	139.96 ± 5.21	12.47 ± 0.45	1.40 ± 0.05
CbR-D	29.58 ± 0.36	26.68 ± 0.22	48.13 ± 1.44	64.67 ± 2.81	48.11 ± 1.93	98.66 ± 2.01	21.02 ± 0.41	2.07 ± 0.17

CbA-M, C. burhia aerial methanol; CbA-D, C. burhia aerial DCM; CbR-M, C. burhia root methanol; CbR-D, C. burhia root DCM.

Data from three repetitions, with mean ± standard deviation. GAE, gallic acid equivalent; QE, quercetin equivalent; TE, trolox equivalent; EDTAE, EDTA equivalent. All values expressed are means ± SD. of three parallel measurements.

Similarly, HPLC-PDA polyphenolic quantification was performed in order to quantify the phenolic standards in the studied extracts and the results are presented in Table 2, while, the HPLC-PDA chromatograms of the quantified phenolics in the tested extracts are given in Supplementary Figures 1, 2. In comparison to the other extracts, *C. burhia* methanol root extract comprised a significant quantity of phenolics (4.28 μg/mg), with the highest amounts of epicatechin (0.71 μg/mg extract) and *p*-coumaric acid (0.68 μg/mg extract), while rutin (0.33 μg/mg extract) was quantified in lesser amount. Likewise, aerial methanol extract presented the highest quantities of epicatechin (1.89 μg/mg extract), while DCM root extract displayed the lowest amounts of carvacrol (0.65 μg/g extract). Both roots and aerial DCM extracts accounted for the least amounts of phenolic standards (0.65 and 0.36 μg/g extract, respectively), which could be due to the extracts being nonpolar. Further investigations of plant extracts/fractions can be done to separate bioactive compounds with potentially important functions as a result of this phenolic profiling.

**TABLE 2:** HPLC-PDA quantification (μg/mg) of phenolics in *C. burhia* samples.

Tested samples	Polyphenolics quantified (μg/mg dry extract)											
	Gallic acid	Catechin	4-OH benzoic acid	Vanillic acid	Epicatechin	Syringic acid	3-OH-4-MeO benzaldehyde	*p*-coumaric acid	Rutin	Naringenin	Carvacrol	Total (μg/mg)
CbA-M	nd	nd	BLD	nd	1.89 ± 0.24	nd	nd	nd	nd	BLD	0.42 ± 0.03	2.32
CbA-D	nd	nd	nd	nd	nd	nd	BLD	nd	nd	nd	0.36 ± 0.03	0.36
CbR-M	0.49 ± 0.04	0.57 ± 0.06	0.51 ± 0.04	0.53 ± 0.05	0.71 ± 0.06	0.45 ± 0.04	nd	0.68 ± 0.07	0.33 ± 0.03	nd	nd	4.28
CbR-D	nd	nd	nd	nd	nd	nd	nd	nd	nd	nd	0.65 ± 0.05	0.65

CbA-M, C. burhia aerial methanol; CbA-D, C. burhia aerial DCM; CbR-M, C. burhia root methanol; CbR-D, C. burhia root DCM.

Nd, not detected; Chlorogenic acid, 3-OH benzoic acid, sinapinic acid, t-ferulic acid, naringin, 2,3-diMeO benzoic acid, benzoic acid, o-coumaric acid were not detected in any of the tested plant extracts.

**FIGURE 1:**
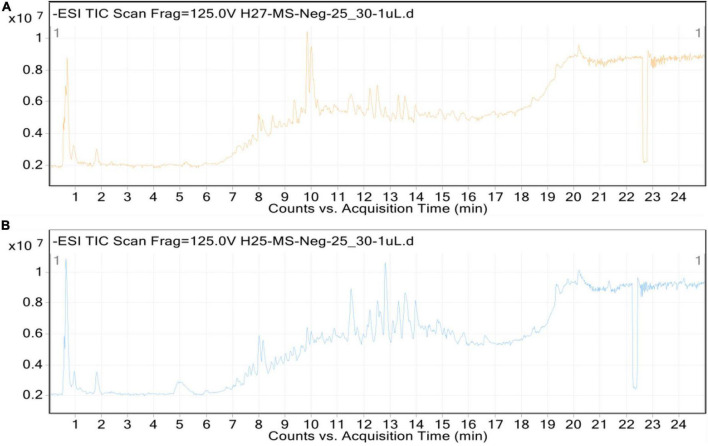
Total ion chromatograms (TICs) of *C. burhia* aerial **(A)** and root **(B)** extracts.

Additionally, methanolic extracts of *C. burhia* roots and aerial parts were subjected to UHPLC-MS analysis in order to get thorough profiles of individual secondary metabolites. Figures 1A,B depict standard total ion chromatograms with mass spectrometric peaks for both extracts. Tables 3, 4 give a preliminary list of secondary metabolites found in aerial and root extracts, respectively. A total of 36 distinct secondary metabolites were detected in the methanolic aerial extract. A preliminary analysis of the root extract identified 53 distinct chemicals. Majority of the compounds belonged to phytoconstituents’ phenols, flavonoid, saponin, coumarin, and glycoside classes. Polyphenols, notably flavonoids and coumarins, have been discovered to possess a wide range of health benefits, including antibacterial, enzyme inhibitory and antioxidant capabilities (Dilworth et al., 2017), whereas glycosides, tannins, alkaloids, and resins have been shown to have antibacterial activities (Rascon-Valenzuela et al., 2017). According to our research, this is the first time this plant has been profiled in such detail.

**TABLE 3:** UPHLC-MS analysis tentative identification of the secondary metabolites from *C. burhia* aerial methanol extract (negative ionization mode).

No.	RT (min)	Mol. mass	Tentative identification	Chemical formula	Compound class	B. peak (*m/z)*
1	0.643	216.0412	Isobergaptene	C_12_ H_8_ O_4_	Coumarin	215.0412
2	7.182	294.1315	Ethyl (S)-3-hydroxybutyrate glucoside	C_12_ H_22_ O_8_	Glycosides	293.1315
4	7.635	640.1647	Isorhamnetin 3-glucosyl-(1- > 6)-galactoside	C_28_ H_32_ O_17_	Flavonoid	639.1647
5	7.747	154.0265	3,4-Dihydroxybenzoic acid	C_7_ H_6_ O_4_	Antioxidant	153.0265
6	7.759	328.0796	Bergenin	C_14_ H_16_ O_9_	Phyto	327.0796
7	7.792	432.1279	Apiosylglucosyl 4-hydroxybenzoate	C_18_ H_24_ O_12_	Glycoside	431.1279
8	8.027	682.1747	Isorhamnetin 3-(6′′′-acetylglucosyl)(1- > 3)-galactoside	C_30_ H_34_ O_18_	Flavonoid	681.1747
9	8.482	226.1206	12-hydroxyjasmonic acid	C_12_ H_18_ O_4_	Carboxylic acid	225.1206
10	8.509	330.1307	(±)-3-(4-Hydroxyphenyl)-1,2-propanediol 4′-O-glucoside	C_15_ H_22_ O_8_	Phenolic glycosides	329.1307
11	8.642	218.1154	3-hydroxy-sebacic acid	C_10_ H_18_ O_5_	Fatty acids	217.1154
13	9.35	286.0482	5,7,2′,3′-Tetrahydroxyflavone	C_15_ H_10_ O_6_	Flavone	285.0482
15	9.864	270.0534	Demethyltexasin	C_15_ H_10_ O_5_	Flavonoid	269.0534
17	10.039	300.064	Kaempferide	C_16_ H_12_ O_6_	Flavone	299.064
19	10.249	200.1047	Decenedioic acid	C_10_ H_16_ O_4_	Fatty acids	199.1047
20	10.42	254.0581	7,4′-Dihydroxyflavone	C_15_ H_10_ O_4_	Flavone	253.0581
21	10.509	286.0479	5,7,2′,3′-Tetrahydroxyflavone	C_15_ H_10_ O_6_	Flavone	285.0479
22	10.917	268.0373	Coumestrol	C_15_ H_8_ O_5_	Phytoestrogens	267.0373
23	11.211	298.0478	8-Methoxycoumestrol	C_16_ H_10_ O_6_	Coumestans	297.0478
24	11.45	624.2635	Kanokoside D	C_27_ H_44_ O_16_	Glycoside	623.2635
26	11.574	314.079	Luteolin 5,3′-dimethyl ether	C_17_ H_14_ O_6_	Flavonoid	313.079
27	11.815	370.1053	Neouralenol	C_20_ H_18_ O_7_	Flavonoid	369.1053
28	11.877	354.1105	2,3-Dehydrokievitone	C_20_ H_18_ O_6_	Iso flavone	353.1105
29	11.883	288.2301	9,16-dihydroxy-palmitic acid	C_16_ H_32_ O_4_	Hydroxy fatty acid	287.2301
30	12.137	562.2627	19-Hydroxycinnzeylanol 19-glucoside	C_26_ H_42_ O_13_	Glycoside	561.2627
34	13.574	452.1087	Cinchonain Ib	C_24_ H_20_ O_9_	Flavonolignan	451.1087
35	14.603	336.0987	Isosojagol	C_20_ H_16_ O_5_	Coumestans	335.0987
36	18.507	272.2352	2-Hydroxyhexadecanoic acid	C_16_ H_32_ O_3_	Fatty acids	271.2352

RT, retention time; B. Peak, base peak.

**TABLE 4:** UPHLC-MS analysis tentative identification of the secondary metabolites from *C. burhia* root methanol extract (negative ionization mode).

No.	RT (min)	Mass	Tentative identification	Chemical formula	Compound class	B. peak (*m/z)*
1	7.794	432.1273	Apiosylglucosyl 4-hydroxybenzoate	C_18_ H_24_ O_12_	Glycoside	431.1273
2	8.287	207.0894	Phenylpropionylglycine	C_11_ H_13_ NO_3_	Acyl glycine	208.0894
3	8.49	462.1168	Tricin 4′-apioside	C_22_ H_22_ O_11_	Flavone	461.1168
4	8.871	416.1103	3′,4′-Dihydroxyflavone 4′-glucoside	C_21_ H_20_ O_9_	Flavone	415.1103
5	9.213	372.1214	7,8,3′,4′,5′-Pentamethoxyflavone	C_20_ H_20_ O_7_	flavone	371.1214
6	9.351	286.0481	5,7,2′,3′-Tetrahydroxyflavone	C_15_ H_10_ O_6_	Flavone	285.0481
7	9.507	370.1056	Neouralenol	C_20_ H_18_ O_7_	Flavone	369.1056
8	9.614	406.0905	5,6,3′,5′-Tetrahydroxy-3,7,8,4′-tetramethoxyflavone	C_19_ H_18_ O_10_	Flavonoids	405.0905
10	9.856	270.0536	Demethyltexasin	C_15_ H_10_ O_5_	Isoflavonoe	269.0536
11	9.942	138.0316	*p*-Salicylic acid	C_7_ H_6_ O_3_	Phenol	137.0316
13	10.034	300.0636	Kaempferide	C_16_ H_12_ O_6_	Flavone	299.0636
14	10.25	200.1051	Decenedioic acid	C_10_ H_16_ O_4_	Phyto	199.1051
15	10.358	584.2616	Pubescenol	C_32_ H_40_ O_10_	Withanolide	583.2616
16	10.424	254.0584	7,4′-Dihydroxyflavone	C_15_ H_10_ O_4_	Flavone	253.0584
17	10.553	284.0683	Texasin	C_16_ H_12_ O_5_	Phyto	283.0683
18	10.756	390.0955	5,7,2′-Trihydroxy-3,6,4′,5′-tetramethoxyflavone	C_19_ H_18_ O_9_	Flavone	389.0955
19	10.822	354.1103	2,3-Dehydrokievitone	C_20_ H_18_ O_6_	Phyto	353.1103
20	10.921	268.0373	Coumestrol	C_15_ H_8_ O_5_	Coumestans	267.0373
21	11.214	454.1632	5,2′,4′,5′-Tetrahydroxy-3-(3-hydroxy-3-methylbutyl)-6″,6″ dimethylpyrano[2″,3″:7,8]flavone	C_25_ H_26_ O_8_	Flavone	453.1632
22	11.217	298.048	8-Methoxycoumestrol	C_16_ H_10_ O_6_	Coumestans	297.048
23	11.293	352.0607	3′-O-Methyl-(-)-epicatechin-5-O-sulfate	C_16_ H_16_ O_7_S	Flavonoids	351.0607
24	11.448	624.2634	Kanokoside D	C_27_ H_44_ O_16_	Terpene glycoside	623.2634
25	11.476	578.2573	Withaperuvin H	C_30_ H_42_ O_9_ S	Withanolide	577.2573
26	11.515	400.116	Torosaflavone A	C_21_ H_20_ O_8_	Flavonoids	399.116
27	11.52	468.1045	Gyrophoric acid	C_24_ H_20_ O_10_	Phyto	467.1045
28	11.561	330.241	5,8,12-trihydroxy-9-octadecenoic acid	C_18_ H_34_ O_5_	Fatty acids	329.241
29	11.63	352.0947	Psoralidin oxide	C_20_ H_16_ O_6_	Coumestans	351.0947
30	11.787	314.0793	Luteolin 5,3′-dimethyl ether	C_17_ H_14_ O_6_	Flavonoids	313.0793
31	12.099	256.0738	6-Demethylvignafuran	C_15_ H_12_ O_4_	Isoflavonoid	255.0738
32	12.141	562.2625	19-Hydroxycinnzeylanol 19-glucoside	C_26_ H_42_ O_13_	Glycosides	561.2625
33	12.638	354.1101	2,3-Dehydrokievitone	C_20_ H_18_ O_6_	Flavanone	353.1101
34	13.249	220.0737	Polygonolide	C_12_ H_12_ O_4_	Coumarins	219.0737
35	13.368	322.1208	5,7-Dihydroxy-8-prenylflavone	C_20_ H_18_ O_4_	Flavone	321.1208
36	13.375	368.1228	Aurmillone	C_21_ H_20_ O_6_	Isoflavonoe	367.1228
37	13.512	438.1681	Morusignin L	C_25_ H_26_ O_7_	Flavones	437.1681
38	13.572	676.2315	Artonin D	C_40_ H_36_ O_10_	Chalcones	675.2315
39	13.573	452.11	Cinchonain Ib	C_24_ H_20_ O_9_	Phyto	451.11
40	13.581	338.1163	(-)-Glyceollin I	C_20_ H_18_ O_5_	Phytoalexins	337.1163
41	13.991	336.1001	Isosojagol	C_20_ H_16_ O_5_	Coumestans	335.1001
42	14.229	440.1835	Exiguaflavanone C	C_25_ H_28_ O_7_	Flavanone	439.1835
43	14.474	354.1102	2,3-Dehydrokievitone	C_20_ H_18_ O_6_	Flavanone	353.1102
44	14.607	450.0928	Exserohilone	C_20_ H_22_ N_2_ O_6_ S_2_	Indoles	449.0928
45	14.721	342.1104	5,7,2′,5′-tetramethoxyflavone	C_19_ H_18_ O_6_	Flavone	341.1104
46	14.82	334.0844	Sophoracoumestan A	C_20_ H_14_ O_5_	Coumeston	333.0844
47	15.079	340.0952	Methylophiopogonone A	C_19_ H_16_ O_6_	Flavonoid	339.0952
48	15.08	324.1364	Isobavachalcone	C_20_ H_20_ O_4_	Chalcones	323.1364
49	15.283	390.1831	Paratocarpin B	C_25_ H_26_ O_4_	Chalcones	389.1831
50	15.494	340.0946	Methylophiopogonone A	C_19_ H_16_ O_6_	Flavonoid	339.0946
51	15.641	406.1783	Honyucitrin	C_25_ H_26_ O_5_	Flavanone	405.1783
52	17.708	296.2354	12-oxo-10Z-octadecenoic acid	C_18_ H_32_ O_3_	Fatty acids	295.2354
53	18.511	272.2355	2-Hydroxyhexadecanoic acid	C_16_ H_32_ O_3_	Fatty acids	271.2355

RT, retention time; B. Peak, base beak.

### Antioxidant potential

Metabolic processes typically produce reactive oxygen species (ROS). Excessive accumulation of ROS causes tissue injury and inflammation by damaging fatty acids, DNA, and proteins. As a result of these illnesses, plant extracts have been examined for their possible function in reducing the oxidative stress burden (Zengin et al., 2022).

Antioxidant activity of *C. burhia* extracts was tested using six different assays, the findings of which may be found in Table 1. To sum up, it was shown that the roots and aerial methanolic extracts had the highest radical scavenging and reducting power assays’ maximum values. Bioactive components with reducing power and anti-oxidant activity have been shown to have a favorable correlation with the amount of phenols and flavonoids found in this extract (Khan et al., 2019). Antioxidant activity was found in phenolic compounds quantified through HPLC-PDA, including 4-OH benzoic acid, vanillic acid, syringaldehyde, *p*-coumaric acid, and carvacrol (Verma et al., 2008). As mentioned in Table 1, the root-methanol extract was the most active for DPPH radical scavenging (50.04 mg TE/g extract) and CUPRAC reducing power potential (139.96 mg TE/g extract). Likewise, the aerial-methanol extract exhibited maximum ABTS radical scavenging (94.05 mg TE/g extract) and FRAP reducing power potential (64.23 mg TE/g extract). The DCM aerial extract exhibited the highest potential for phosphomolybdenum assay at 60.46 mg TE/g and metal chelation activity at 2.24 mg EDTAE/g. Previous studies have shown that this plant has significant antioxidant activity which validates our current findings (Talaviya et al., 2014; Ahmed, 2018). Rutin and naringenin, two important flavonoids with antioxidant potential, were also found in the current study’s HPLC polyphenol quantification and UHPLC-MS analysis (Yang et al., 2008; Cavia-Saiz et al., 2010).

### Enzyme inhibition activities

Enzyme inhibition is gaining popularity as a therapeutic technique for various global health challenges, including type 2 diabetes, neurodegenerative diseases, and dermatological disorders. This phenomenon illustrates the strategy of inhibiting certain enzymes from treating specific diseases. Neurodegenerative diseases like Alzheimer’s and Parkinson’s have been linked to butyrylcholinesterase (BChE) and Acetylcholinesterase (AChE) (Zengin et al., 2018). Some research has shown that isolated compounds and plant extracts can both inhibit cholinesterase activity (Ballard et al., 2005). Galantamine, an alkaloid extracted from the *Galanthus woronowii* plant, is one example. Treatment for mild to moderate Alzheimer’s disease with the AChE inhibitor galantamine (Colovic et al., 2013). Previously, significant AChE inhibition potential has been reported in ethanolic extract of *C. hebecarpa* leaves (IC_50_: 208.6 μg/mL) (Rao et al., 2017). As presented in Table 5, the aerial DCM aerial showed maximum inhibition for AChE (4.01 mg GALAE/g extract) and BChE (4.28 mg GALAE/g extract). While, DCM root extract and methanolic aerial extract displayed the lowest inhibition potential against AChE and BChE (2.07 and 2.93 mg GALAE/g extract), respectively.

**TABLE 5:** Enzyme inhibition effects of *C. burhia* aerial and root extracts.

Extracts	AChE (mg GALAE/g extract)	BChE (mg GALAE/g extract)	Tyrosinase (mg KAE/g extract)	Amylase (mmol ACAE/g extract)	Glucosidase (mmol ACAE/g extract)
CbA-M	3.79 ± 0.27	2.93 ± 0.07	131.72 ± 0.52	0.63 ± 0.03	1.86 ± 0.04
CbA-D	4.01 ± 0.41	4.28 ± 0.19	124.95 ± 0.35	0.67 ± 0.02	1.92 ± 0.01
CbR-M	3.29 ± 0.34	3.37 ± 0.12	128.51 ± 1.35	0.60 ± 0.01	1.89 ± 0.01
CbR-D	2.07 ± 0.16	3.22 ± 0.24	120.76 ± 0.40	0.70 ± 0.03	na

GALAE, galatamine equivalent; KAE, kojic acid equivalent; ACAE, acarbose equivalent; na, not active. All values expressed are means ± S.D. of three parallel measurements.

The enzyme tyrosinase catalyzes human melanin biosynthesis, also known as melanogenesis, a physiological process that results in the production of melanin (Muddathir et al., 2017). Considering that the inhibition of tyrosinase activity can control melanin formation, dermatological conditions, such as those characterized by excessive melanin pigmentation, could benefit from tyrosinase inhibitor treatment (Jdey et al., 2017). Tyrosinase inhibition can also be used in the food industry. Fruits and vegetables can gain a lot from the inhibition of tyrosinase. Enzyme tyrosine catalyzes the decomposition of phenolic compounds, which results in undesirable color and taste (Zaidi et al., 2014). *C. burhia* methanol aerial extract showed maximum tyrosinase inhibition, i.e., 131.72 mg KAE/g extract. In comparison, the methanolic root extract showed inhibition of 128.51 mg KAE/g extract, followed by DCM aerial and DCM root extracts124.95 and 120.76 mg KAE/g extract, respectively (Table 5). According to previous studies, different phenolics and flavonoids have been shown to have anti-tyrosinase properties, which may explain why the methanolic extract rich in phenolic and flavonoid compounds was found active against mushroom tyrosinase (Zielinska et al., 2017; Choi et al., 2021). Significant tyrosinase inhibition potential of ethanolic extract of another *Crotalaria* species *C. hebecarpa* (IC_50_: 40.15 μg/mL), has been reported previously (Rao et al., 2017). Similarly, another study reported the methanol and aqueous extracts of *C. juncea* shoots to show moderated tyrosinase inhibition (16.12 and 22.45%) at 1 mg/mL (Ketprayoon and Chaicharoenpong).

Hyperglycemia occurs when the pancreas produces less insulin or the cells’ insulin sensitivity decreases. According to the World Health Organization, approximately 422 million individuals worldwide have been diagnosed with diabetes. Although synthetic medications have advanced, the number of people with diabetes continues to rise at an alarming rate. Several medicinal herbs, including curcumin, have been demonstrated to be beneficial in the diabetes (Choudhury et al., 2018; Obih et al., 2019). The alpha-amylase and alpha-glucosidase inhibitors acarbose, miglitol, and viglibose have been established. Acarbose is derived from plants. Bloating, flatulence, and other gastrointestinal discomforts have been linked to an excess inhibition of -amylase (Figueiredo-González et al., 2016). As a result, the mild inhibition of α-amylase and the significant inhibition of α-glucosidase were preferred (Kazeem et al., 2013).

In light of these findings, the enzyme inhibition capability of *C. burhia* extract and fractions was assessed against the clinically significant enzymes involved in diabetes, namely α-glucosidase and α-amylase. The current investigations have revealed (Table 5) that *C. burhia* extracts a mild inhibitor of α-glucosidase and α-amylase enzymes. The DCM root extract displayed the highest inhibitory potential against α-amylase (0.70 mmol ACAE/g extracts) while DCM aerial extract presented maximum potential against α-glucosidase (1.92 mmol ACAE/g extracts). The α-amylase inhibition results of *C. burhia* extracts were ordered as follows: CbR-D > CbA-D > CbA-M > CbR-M.

### Cytotoxic activity

Two breast cancer cell lines, MCF-7 and MDA-MD-231, were tested for cytotoxicity of *C. burhia* extracts, as shown in Table 6. The results show that none of the extracts presented significant toxicity to the breast cell line used in the study. For MCF-7 and MDA-MB-231 cell lines, the CbA-M extract was found to be the most effective, with a percentage viability of 74.29 and 70%, respectively. Likewise, the CbR-M extract was also found to be considerably active against the MDA-MB-231 cell line, likewise, the CbA-D extract was also active against this cell line. The CbR-D extract was less toxic to either of the cell lines that were tested. *In-vivo* toxicity studies are recommended following this preliminary toxicity testing of the plant extract studied.

**TABLE 6:** Cytotoxicity of *C. burhia* samples against breast cell lines.

Extracts	% Viability (200 μg/mL)	
	MCF-7	MDA-MB-231
CbA-M	74.29	70.56
CbA-D	4.0297	61.06
CbR-M	23.98	84.04
CbR-D	14.546	2.69

CbA-M, C. burhia aerial methanol; CbA-D, C. burhia aerial DCM; CbR-M, C. burhia root methanol; CbR-D, C. burhia root DCM. Data from three repetitions, with mean ± standard deviation.

### Principal component analysis

**FIGURE 2:**
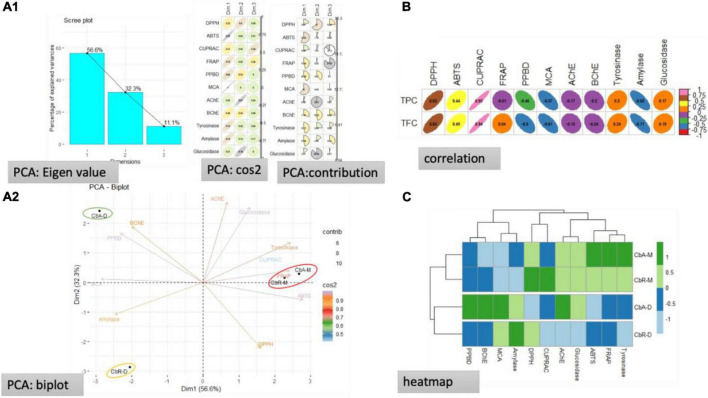
Statistical evaluations, **(A1)** eigenvalues and percentage of variability expressed by the factors; **(A2)** representation of biological activities on the correlation circle based on PCA; **(B)** correlation coefficients between total bioactive compounds and biological activities [Pearson Correlation Coefficient (R), *p <* 0.05]; **(C)** heat map of extracts in according to bioactive compounds and biological activities.

Data from multiple tests can be analyzed using PCA. To accomplish this, we used PCA to analyze the tested extracts. Correlation, clustering, and PCA were used to show how aerial and root extracts interacted with the biological assays. The results are summarized in Figure 2. Three dimensions summarizing, respectively, 50.6, 32.3, and 11.1% of the biological activities variability were obtained (Figure 2A1). It was noted that the two principal components were built by PCA, explaining 88.9% of the total variability, with dimension 1 (56.6%) and dimension 2 (32.3%) (Figure 2A2). Moreover, it was seen that the variables DPPH, ABTS, CUPRAC, tyrosinase, glucosidase, and AChE were strongly associated with the origination of axis 1 (56.6%), whereas, the variables inclusive of amylase, phosphomolybdenum, and BChE were strongly contributed to the formation of axis 2 (32.3%). The TPC was noted to be highly positive co-related with the CUPRAC, while a positive moderate co-relation was noted for the DPPH, and ABTS activities, whereas, a weak positive relationship was observed for the tyrosinase and glucosidase. Likewise, a moderate to weak negative correlation was observed among TPC and FRAP, PPBD, MCA, AChE, and BChE, while a strong negative co-relation occurred for the TPC and amylase. Similarly, the TFC presented a considerable positive relationship for CUPRAC, DPPH, ABTS, moderate to weak positive correlation for the tyrosinase, glucosidase, and FRAP, and a weak relationship for the PPBD, MCA, and amylase. These results are further verified from the heatmap.

## Conclusion

The specific phytochemical and biological composition of several extracts of the *C. burhia* plant has emphasized the possible consequences of these extracts. Secondary metabolites in the phenolic, flavonoid, and glycoside classes were identified through HPLC-PDA and UHPLC-MS analysis. It was found that the most polar solvent extracts had the highest bioactive content. All of the tested extracts had varying antioxidant and enzyme-inhibiting potential. In addition, statistical studies confirm the link between the contents and the apparent biological activities. *C. burhia* plant extracts can be used as a natural source of bioactive compounds, according to the findings of this comprehensive report. However, more exploration is required for better insight in terms of isolation and characterization studies.

## Data availability statement

The original contributions presented in this study are included in the article/Supplementary material, further inquiries can be directed to the corresponding author/s.

## Author contributions

SA, HS, and UK: writing and editing. MF, IP, NAk, KAm, and MH: data curation. KAl, FA, MS, ML, and NAh: supervision. All authors contributed to the article and approved the submitted version.
