# Supplementary material for: A comprehensive phytochemical, biological, and toxicological studies of roots and aerial parts of Crotalaria burhia Buch.-Ham: An important medicinal plant
Source: Front Plant Sci. 2022 Sep 16;13:988352. doi: 10.3389/fpls.2022.988352 (PMC9533709; doi:10.3389/fpls.2022.988352)
Supplement: Supplementary file 1 [file Data_Sheet_1.docx]

**Supplementary:**

**A comprehensive Phytochemical, biological, and toxicological studies of roots and aerial parts of *Crotalaria burhia* Buch.-Ham: An important medicinal plant**

Sirajudheen Anwar^1^*, Muhammad Faisal Nadeem^2^, Irfan Pervaiz^3^, Umair Khurshid^4^, Khurram Amir^5^, Muhammad Haseeb ur Rehman^5,6^, Khaled Almansour^7^, Farhan Alshammari^7^, Mohd Farooq Shaikh^9^, Marcello Locatelli^9^, Nafees Ahemad^10^, Hammad Saleem^2^*

*^1^Department of Pharmacology and Toxicology, College of Pharmacy, University of Hail, Hail, Saudi Arabia*

*^2^Institute of Pharmaceutical Sciences (IPS), University of Veterinary & Animal Sciences (UVAS), Lahore, Pakistan,*

*^3^Department of Pharmacy, The University of Chenab, Gujrat, Pakistan*

*^4^Department of Pharmaceutical Chemistry, Faculty of Pharmacy, The Islamia University of Bahawalpur, Pakistan*

*^5^Akhtar Saeed College of Pharmacy, Canal Campus, Lahore Pakistan*

*^6^Government College University, Faisalabad, Faisalabad, Pakistan*

*^7^Department of Pharmaceutics, College of Pharmacy, University of Hail, Hail, Saudi Arabia.*

*^8^Neuropharmacology Research Strength, Jeffrey Cheah School of Medicine and Health Sciences,*

*^9^Department of Pharmacy, University ‘G. d’Annunzio” of Chieti-Pescara, 66100, Chieti, Italy*

*^10^School of Pharmacy, Monash University Malaysia, Bandar Sunway 47500, Selangor, Malaysia.*

**Corresponding author:**

Sirajudheen Anwar ([si.anwar@uoh.edu.sa](mailto:si.anwar@uoh.edu.sa) );

Hammad Saleem (hammad.saleem@uvas.edu.pk)

**Abstract:**

This study was designed to seek the phytochemical analysis, antioxidant, enzyme inhibition, and toxicity potentials of methanol and dichloromethane (DCM) extracts of aerial and root parts of *Crotalaria burhia*. Total bioactive content, high-performance liquid chromatography-photodiode array detector (HPLC-PDA) polyphenolic quantification, and ultra-high performance liquid chromatography-mass spectrometry (UHPLC-MS) analysis were utilized to evaluate the phytochemical composition. Antioxidant (including 2,2-diphenyl-1-picryl-hydrazyl-hydrate (DPPH), 2,2'-azino-bis(3-ethylbenzothiazoline-6-sulfonic acid (ABTS), ferric reducing antioxidant power assay (FRAP), cupric reducing antioxidant capacity CUPRAC, phosphomolybdenum, and metal chelation assays) and enzyme inhibition ( against acetylcholinesterase (AChE), butyrylcholinesterase (BChE), α-glucosidase, α-amylase, and tyrosinase) assays were carried out for biological evaluation. The cytotoxicity was tested against MCF-7 and MDA-MB-231 breast cell lines. The root-methanol extract contained the highest levels of phenolics (37.69 mg gallic acid equivalent/g extract) and flavonoids (83.0 mg quercetin equivalent/g extract) contents, and was also the most active for DPPH (50.04 mg Trolox equivalent/g extract) and CUPRAC (139.96 mg Trolox equivalent /g extract) antioxidant assays. Likewise, the aerial-methanol extract exhibited maximum activity for ABTS (94.05 mg Trolox equivalent/g extract) and FRAP (64.23 mg Trolox equivalent/g extract) assays. The aerial-DCM extract was noted to be a convincing cholinesterase (AChE; 4.01 and BChE; 4.28 mg galantamine equivalent/g extract), and α-glucosidase inhibitor (1.92 mmol acarbose equivalent/g extract). All of the extracts exhibited weak to modest toxicity against the tested cell lines. A considerable quantities of gallic acid, catechin, 4-OH benzoic acid, syringic acid, vanillic acid, 3-OH-4-MeO benzaldehyde, epicatechin, *p*-coumaric acid, rutin, naringenin, and carvacrol were quantified *via* HPLC-PDA analysis. UHPLC-MS analysis of methanolic extracts from roots and aerial parts revealed the tentative identification of important phytoconstituents such as polyphenols, saponins, flavonoids, and glycoside derivatives. To conclude, this plant could be considered a promising source of origin for bioactive compounds with several therapeutic uses.

**Keywords:** *Crotalaria burhia*; secondary metabolites; antioxidants; enzyme inhibition; toxicity

1. ***Phytochemical Composition***
   1. *Total phenolic and Flavonoid content*

Total phenolic and flavonoid contents were evaluated in the all crude extract as described previously (Bahadori et al., 2017;Grochowski et al., 2017) by means of a well-known procedure as follow.

For the total phenolic content, 0.25 mL of the sample solution (1 mg/mL) was mixed with the diluted (1:9, v/v) Folin–Ciocalteu reagent (1 mL). 0.75 mL of a Na_2_CO_3_ solution (1%) was added after 3 min and then the sample absorbance was read at 760 nm after 2 h of incubation at room temperature.

For the total flavonoid content, 1 mL of the sample solution (1 mg/mL) was mixed with an equal volume of aluminium chloride (2%) solution in methanol and the absorbance was read at 415 nm after 10 min of incubation at room temperature. The outcomes of total phenolic constituents were reported as equivalents of gallic acid (mg GAE/g extract) while the results of total flavonoid constituents were recorded as equivalents of rutin (mg RE/g extract).

- 1. *UHPLC-MS-Instrumentation*

Secondary metabolites were evaluated by RP-UHPLC-MS. UHPLC of Agilent 1290 Infinity LC system coupled to Agilent 6520 Accurate-Mass Q-TOF mass spectrometer with dual ESI source was used. Column specifications were as: Agilent Zorbax Eclipse XDB-C18, narrow-bore 2.1 x 150 mm, 3.5 micron (P/N: 930990-902). Column and auto-sampler temperature were maintained at 25 °C and 4 °C, respectively. Flow rate was 0.5 mL/min. Mobile phases used were: A - 0.1% formic acid in water, B - 0.1% formic acid in acetonitrile. Injection volume was 1.0 µL. Run time was 25 min and post-run time was 5 min. Full scan MS analysis was done over a range of *m/z* 100-1000 using electrospray ion source in negative mode. Nitrogen was supplied as nebulizing and drying gas at flow rates of 25 and 600 L/hour, respectively. The drying gas temperature was 350 °C. The fragmentation voltage was optimized to 125 V. Analysis was performed with a capillary voltage of 3500 V. Data was processed with Agilent Mass Hunter Qualitative Analysis B.05.00 (Method: Metabolomics-2017- 00004.m). Identification of compounds was done from Search Database: METLIN_AM_PCDL-N- 170502.cdb, with parameters as: Match tolerance: 5 ppm, Positive Ions: +H, +Na, +NH_4_, Negative Ions: -H (Saleem et al., 2019).

- 1. *HPLC-PDA polyphenolic quantification*
     1. *HPLC conditions*

HPLC analyses were performed on a Waters liquid chromatograph equipped with a model 600 solvent pump and a 2996 photodiode array detector, and Empower v.2 Software (Waters Spa, Milford, MA, USA) was used for acquisition of data. A C18 reversed-phase packing column (Prodigy ODS (3), 4.6 × 150 mm, 5 μm; Phenomenex, Torrance, CA, USA) was used for the separation and the column was thermostated at 30±1°C using a Jetstream2 Plus column oven. The UV/Vis acquisition wavelength was set in the range of 200–500 nm. The quantitative analyses were achieved at maximum wavelength for each compounds. The injection volume was 20 μL. The mobile phase was directly *on-line* degassed by using Biotech DEGASi, mod. Compact (LabService, Anzola dell’Emilia, Italy). Gradient elution was performed using the mobile phase water-acetonitrile (93:7, v/v, 3% acetic acid) as reported in the literature (Locatelli et al., 2017;Di Sotto et al., 2018). All the prepared sample solutions were centrifuged and the supernatant was injected into HPLC.

- - 1. *Preparation of standard solutions and samples*

The stock solutions of phenolics were made at a concentration of 1 mg/mL in a final volume of 10 mL of methanol. Working solutions of mixed standards at the concentrations of 10, 25, 50, 75, 100, 150 and 200 μg/mL were made by dilution of stock solution in volumetric flasks with the mobile phase. Then the standards were injected into the HPLC-UV/Vis system. Working solutions of mixed standards at the concentrations of 0.25, 0.5, 1, 2.5, 5, 10, and 20 μg/mL were made by dilution of stock solution in volumetric flasks with the mobile phase. Then the standards were injected into the HPLC-UV/Vis system. Each solid sample was weighted and solubilized in mobile phase in 1:1 (*w:v*) ratio. In this case, the obtained concentrations (μg/mL) correspond to the total amount (μg/mg). After solubilization, the sample was centrifuged at 12000 x *g* before HPLC injection. Into the tables, BLD<0.1 μg/mL while BLQ<0.2 μg/mL.

1. ***Antioxidant assays***

The free radical scavenging (DPPH, ABTS), reducing power (FRAP, CUPRAC) phosphomolybdenum (total antioxidant capacity), and metal chelating (ferrous ion chelation) were evaluated following the previous methods as described by Grochowski et al. (2017) (Grochowski et al., 2017).

- 1. *Free radical scavenging activity (DPPH)*

1 mL of the sample solution was added to the DPPH solution (0.267 mM 4 mL, 0.004% methanol solution), and after 30 min of incubation, the absorbance was recorded at 517 nm. Milligrams of Trolox equivalents per gram of dry extract (TE/g extract) were the measure unit.

- 1. *ABTS radical cation scavenging activity*

ABTS^+^ radical cation was obtained following the reaction between 7 mM ABTS solution and 2.45 mM potassium persulfate. 1 mL of the test solution was mixed with 2 mL of ABTS solution and after 30 min the absorbance was recorded at 734 nm. The results were expressed as milligrams of Trolox equivalents per gram of dry extract (TE/g extract).

*2.3. Phosphomolybdenum method*

0.3 mL of the sample solutions were mixed with 3 mL of reagent solution (0.6 M sulfuric acid, 28 mM sodium phosphate and 4 mM ammonium molybdate), and after 90 min the absorbance was recorded at 695 nm. Millimoles of Trolox equivalents per gram of dry extract (TE/g extract) were the measurement unit.

*2.4. Cupric ion reducing (CUPRAC) method*

0.5 mL of the sample solutions were mixed with [CuCl2 (1 mL, 10 mM), neocuproine (1 mL, 7.5 mM), NH_4_Ac buffer (1 mL, 1 M, pH 7.0)] and after 30 min the absorbance was recorded at 450 nm. Milligrams of Trolox equivalents per gram of dry extract (TE/g extract) were the measurement unit.

*2.5. Ferric reducing antioxidant power (FRAP) method*

0.1 mL of the sample solution was added to reagent (2 mL) in acetate buffer (0.3 M, pH 3.6), 2,4,6-tris(2-pyridyl)-s-triazine (TPTZ) (10 mM) in 40 mM HCl and ferric chloride (20 mM) in a final ratio of 10:1:1 (v/v/v) and the absorbance was recorded at 593 nm 30 min. Milligrams of Trolox equivalents per gram of dry extract (TE/g extract) were the measurement unit.

*2.6. Metal chelating activity on ferrous ions*

2.0 mL of the sample solution were added to 0.05 mL of a solution of FeCl_2_ (2 mM), then the reaction was initiated using 0.2 mL of ferrozine (5 mM). After 10 min the absorbance was recorded at 562 nm and the results expressed as milligrams of EDTA equivalents per gram of dry extract (EDTAE/g extract).

1. ***Enzyme inhibition assays***

The possible enzyme inhibitory capacity of all the concentrates against acetylcholinesterase (AChE), butyrylcholinesterase (BChE), tyrosinase, and α-amylase were explored utilizing earlier standard *in-vitro* bio-assays (Grochowski et al., 2017).

- 1. *Cholinesterase*

The reaction mixture composed by the sample solution (50 μL), DTNB (3 mM 125 μL) and enzyme solution (0.265 u/mL AChE or 0.026 u/mL BChE) solution (25 μL) in Tris-HCl buffer (pH 8.0) was added to the substrates [acetylthiocholine iodide (15 mM ATCI) or butyrylthiocholine chloride (1.5 mM BTCl, 25 μL)]. After 15 min of incubation, the absorbance was recorded at 405 nm and the results expressed as milligrams of galantamine equivalents per gram of dry extract (GALAEs/g extract).

- 1. *α-Amylase*

The reaction mixture composed by 25 μL of the sample solution and 50 μL of the α-amylase solution (10 u/mL) in phosphate buffer (pH 6.9 with 6 mM sodium chloride) was added to 50 μL of the starch solution (0.05%) and the reaction was stopped with the addition of 25 μL of HCl (1 M). Then 100 μL of the iodine-potassium iodide solution was added. After 10 min of incubation, the absorbance was recorded at 630 nm and the results expressed as millimoles of acarbose equivalents per gram of dry extract (ACAEs/g extract).

- 1. *Tyrosinase*

25 μL of the sample solution were added to a 40 μL of tyrosinase solution (200 u/mL) and phosphate buffer (40 mM, 100 μL, pH 6.8) in a 96-well microplate and incubated for 15 min at 25 °C. Then the reaction was initiated by adding l-DOPA (10 mM, 40 μL). After 10 min of incubation at room temperature, the absorbance was recorded at 492 nm and the results expressed as milligrams of kojic acid equivalents per gram of dry extract (KAE/g extract).


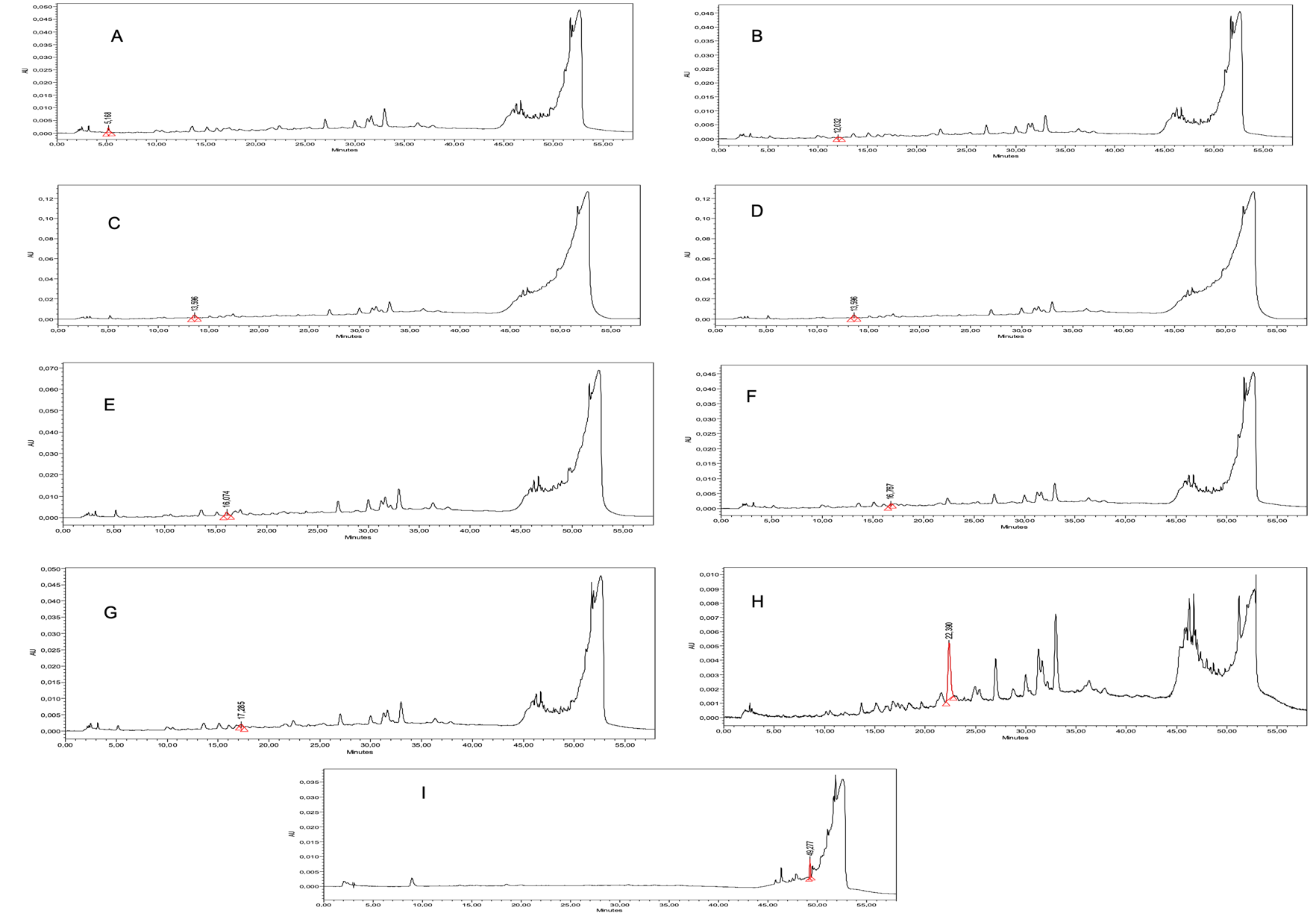


**Figure S1:** HPLC-PDA chromatograms of the quantified phytochemicals in aerial methanol (**A**) gallic acid; (**B**) catechin; (**C**) 4-OH benzoic acid; (**D**) vanillic acid; (**E**) epicatechin; (**F**) syringic acid; (**G**) *p*-coumaric acid; (**H**) rutin, and aerial DCM (**I**) Carvacrol extracts.

**
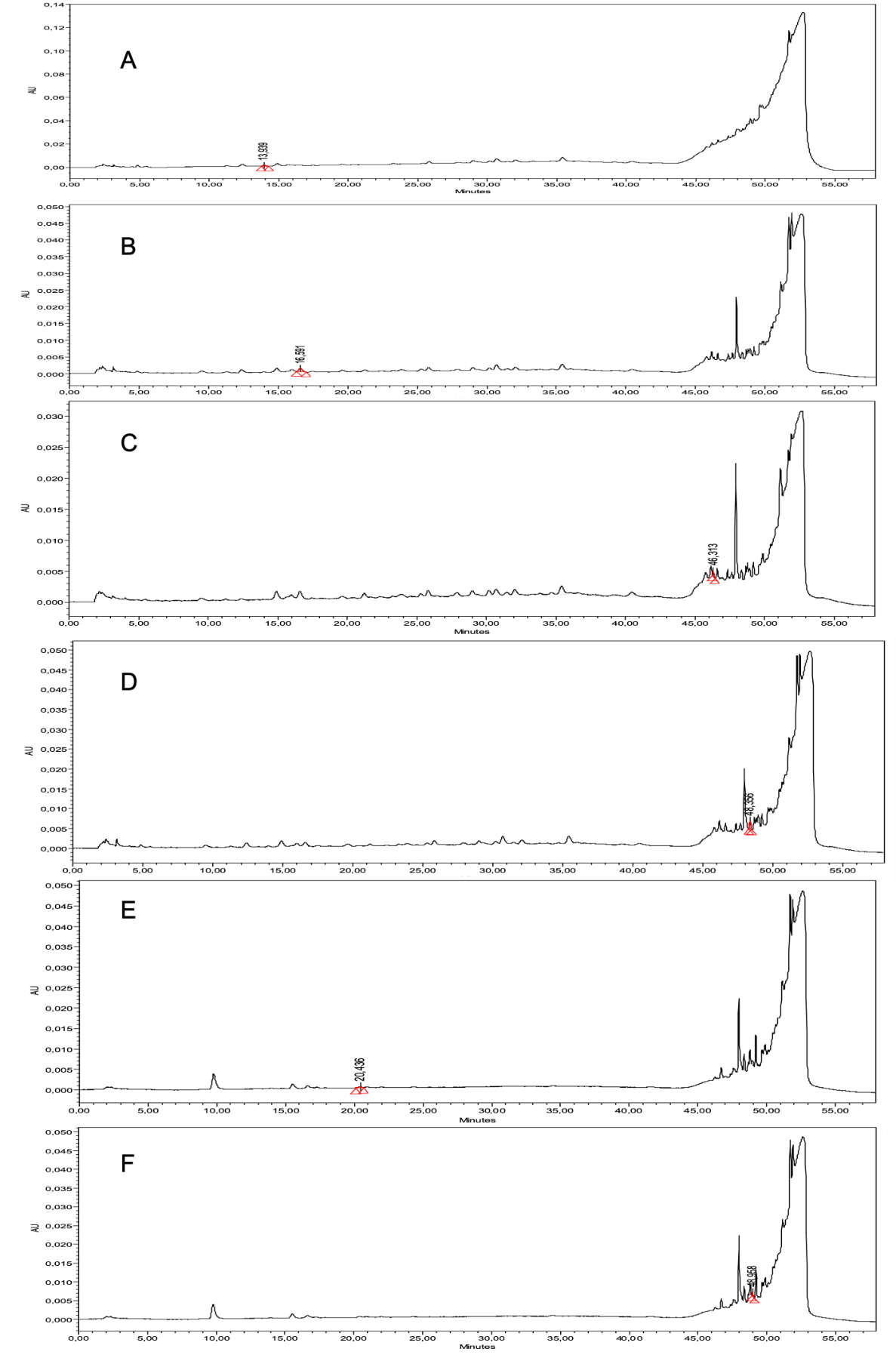
**

**Figure S2:** HPLC-PDA chromatograms of the quantified phytochemicals in root methanol extracts (A) 4-OH benzoic acid; **(B)** 4-OH benzoic acid; **(C)** naringenin; **(D)** carvacrol, and root DCM extracts **(E)** 3-OH-4-MeO benzaldehyde; **(F)** carvacrol.

**Table S1**: Gradient profile

| Time (min) | % water (3% acetic acid) | % acetonitrile (3% acetic acid) | Flow (mL/min) |
| --- | --- | --- | --- |
| 0 | 93 | 7 | 1 |
| 0.1 | 93 | 7 |  |
| 30 | 72 | 28 |  |
| 38 | 75 | 25 |  |
| 45 | 2 | 98 |  |
| 47 | 2 | 98 |  |
| 48 | 93 | 7 |  |
| 58 | 93 | 7 |  |

**Table S2**: Calibration parameter

|  | **Slope** | **Intercept** | **r^2^** | **Wavelength** | **Ret. time (min±S.D.)** |
| --- | --- | --- | --- | --- | --- |
| Gallic acid | 44126 | 1005 | 0.9858 | 271 nm | 4.40±0.06 |
| Catechin | 7022 | 972.4 | 0.9361 | 278 nm | 11.9±0.27 |
| Chlorogenic acid | 22136 | 2475 | 0.9650 | 324 nm | 12.7±0.23 |
| p-OH benzoic acid | 108088 | -774.6 | 0.9504 | 256 nm | 13.2±0.20 |
| Vanillic acid | 51494 | 1141 | 0.9665 | 260 nm | 15.7±0.19 |
| Epicatechin | 8841 | 838.9 | 0.9542 | 278 nm | 16.4±0.23 |
| Syringic acid | 21596 | 369.6 | 0.9672 | 274 nm | 16.7±0.17 |
| 3-OH benzoic acid | 17280 | -1815 | 0.9541 | 295 nm | 16.9±0.21 |
| 3-OH-4-MeO benzaldehide | 60154 | -187.5 | 0.9882 | 275 nm | 20.4±0.18 |
| p-coumaric acid | 96213 | 4217 | 0.9416 | 309 nm | 22.0±0.19 |
| Rutin | 35896 | 1420 | 0.9429 | 256 nm | 24.0±0.14 |
| Sinapinic acid | 498115 | -85290 | 0.9737 | 324 nm | 24.5±0.16 |
| t-ferulic acid | 63520 | -1361 | 0.9969 | 315 nm | 26.1±0.14 |
| Naringin | 22064 | -769 | 0.9964 | 285 nm | 28.4±0.13 |
| 2,3-diMeO benzoic acid | 7163 | -579.3 | 0.9592 | 299 nm | 28.7±0.14 |
| Benzoic acid | 12500 | 842.4 | 0.9901 | 275 nm | 29.4±0.14 |
| o-coumaric acid | 111357 | -4293 | 0.9944 | 276 nm | 30.2±0.16 |
| Quercetin dihydr | 74660 | -6774 | 0.9837 | 367 nm | 39.9±0.24 |
| Harpagoside | 48111 | 1000000 | 0.9901 | 280 nm | 41.2±0.35 |
| t-cinnamic acid | 168680 | -6103 | 0.9933 | 276 nm | 42.7±0.23 |
| Naringenin | 40329 | 305.8 | 0.9577 | 290 nm | 46.0±0.04 |
| Carvacrol | -25903 | 91780 | 0.9785 | 275 nm | 49.5±0.54 |

**References:**

Bahadori, M.B., Asghari, B., Dinparast, L., Zengin, G., Sarikurkcu, C., Abbas-Mohammadi, M., and Bahadori, S. (2017). Salvia nemorosa L.: A novel source of bioactive agents with functional connections. *LWT* 75**,** 42-50.

Di Sotto, A., Di Giacomo, S., Amatore, D., Locatelli, M., Vitalone, A., Toniolo, C., Rotino, G.L., Lo Scalzo, R., Palamara, A.T., and Marcocci, M.E. (2018). A polyphenol rich extract from Solanum melongena L. DR2 peel exhibits antioxidant properties and anti-herpes simplex virus type 1 activity in vitro. *Molecules* 23**,** 2066.

Grochowski, D.M., Uysal, S., Aktumsek, A., Granica, S., Zengin, G., Ceylan, R., Locatelli, M., and Tomczyk, M. (2017). In vitro enzyme inhibitory properties, antioxidant activities, and phytochemical profile of Potentilla thuringiaca. *Phytochemistry Letters* 20**,** 365-372.

Locatelli, M., Zengin, G., Uysal, A., Carradori, S., De Luca, E., Bellagamba, G., Aktumsek, A., and Lazarova, I. (2017). Multicomponent pattern and biological activities of seven Asphodeline taxa: potential sources of natural-functional ingredients for bioactive formulations. *Journal of enzyme inhibition and medicinal chemistry* 32**,** 60-67.

Saleem, H., Htar, T.T., Naidu, R., Nawawi, N.S., Ahmad, I., Ashraf, M., and Ahemad, N. (2019). Biological, chemical and toxicological perspectives on aerial and roots of Filago germanica (L.) huds: Functional approaches for novel phyto-pharmaceuticals. *Food and Chemical Toxicology* 123**,** 363-373.
